# 

**Published:** 1842-12

**Authors:** 


### CONTENTS.

### LIBRARY.

PAGE.

Researches on the Development, Structure and Diseases of the Teeth. By Alexander Nasmyth, F. L. S. F. G. S. .................... 1

### JOURNAL.

Article I.—A Treatise on Mechanical Dentistry. By Solyman Brown, M. D., D. D. S., .................... 81

Article II.—Dissertation on the Diseases of the Maxillary Sinus. By Chapin A. Harris, M. D., D. D. S., .................... 98

Article III.—Contributions to Operative and Mechanical Dentistry. By W. H. Elliot, .................... 133

Article IV.—Osteo-Sarcoma of Lower Jaw—Amputation—Cure. By Charles Bell Gibson, M. D., .................... 138

Article V.—Excision of the Upper Maxillary Bone. By R. D. Mussey, M. D. &c., .................... 142

### BIBLIOGRAPHICAL NOTICES.

Handbuch der Zahnheilkunde, etc. Von C. J. Linderer, Sen., ..... 144

Nouveau Traite Theorique et Pratique de l'Art du Dentiste. Par J. Lefoulon, .................... 144

### DENTAL WORK IN THE PRESS.

Maury's Dental Surgery, .................... 145

### MISCELLANEOUS NOTICES.

Virginia Society of Surgeon Dentists .................... 145

Extract from the Minutes of a Convention of Surgeon Dentists, assembled in the City of Richmond, Va., .................... 146

Anonymous Communications, .................... 148

Dr. Eleazar Parmly's Likeness, .................... 149

Our Reprint, .................... 149

C. Starr Brewster, M. D., D. D. S. .................... 149

More Honours, .................... 150

Another Anomalous Tooth, .................... 150

Osseous union of Deciduous Teeth, .................... 150

Remedies for Tooth-ache, .................... 151

Dr. A. Rostang, .................... 151

Baltimore College of Dental Surgery—Spring Lectures, .................... 151

Formation of a Western Society of Dental Surgeons contemplated, 152

Mania from Decayed Teeth, .................... 152

Dr. James Taylor, .................... 152

